# The TLR4 D299G and T399I SNPs Are Constitutively Active to Up-Regulate Expression of Trif-Dependent Genes

**DOI:** 10.1371/journal.pone.0111460

**Published:** 2014-11-03

**Authors:** Georgina L. Hold, Susan Berry, Karin A. Saunders, Janice Drew, Claus Mayer, Heather Brookes, Nick J. Gay, Emad M. El-Omar, Clare E. Bryant

**Affiliations:** 1 Division of Applied Medicine, Aberdeen University, Aberdeen, United Kingdom; 2 Rowett Institute of Nutrition and Health, Aberdeen University, Aberdeen, United Kingdom; 3 Biomathematics & Statistics Scotland, Aberdeen, United Kingdom; 4 Department of Veterinary Medicine, University of Cambridge, Cambridge, United Kingdom; 5 Department of Biochemistry, University of Cambridge, Cambridge, United Kingdom; SRI International, United States of America

## Abstract

Dysregulated Toll-Like Receptor (TLR) signalling and genetic polymorphisms in these proteins are linked to many human diseases. We investigated TLR4 functional variants D299G and T399I to assess the impact on LPS-induced responsiveness in comparison to wild-type TLR4. The mechanism by which this occurs in unclear as these SNPs do not lie within the lipid A binding domain or dimerisation sites of the LPS-TLR4/MD2 receptor complexes. Transfection of TLR4D299G, TLR4T399I or TLR4D299G. T399I into HEK cells resulted in constitutive activation of an NF-κB reporter gene and a blunting of the LPS-induced reporter activation compared to WT-TLR4. Unstimulated human monocyte/macrophages, from patients with the D299G and T399I SNPs demonstrated a downregulation of many genes, particularly Tram/Trif signalling pathway constitutents compared to the TLR4 wild-type subjects supporting the concept of basal receptor activity. Monocyte/macrophages from carriers of the TLR4 D299G and T399I polymorphisms stimulated with LPS showed >6 fold lower levels of NF-κB and ∼12 fold higher IFN-β gene expression levels compared to wild-type subjects (P<0.05; MWU test) and dramatically altered resultant cytokine profiles. We conclude that these TLR4 SNPs affect constitutive receptor activity which impacts on the hosts ability to respond to LPS challenge leading to a dysregulated sub-optimal immune response to infection.

## Introduction

Dysregulated Toll-Like Receptor (TLR) signalling and genetic polymorphisms in these proteins are linked to many human diseases [1], [2]. A functional polymorphism that alters the amino acid at position 299 in TLR4, the lipopolysaccharide (LPS) receptor, (*TLR4* Asp299Gly - rs4986790) [3], [4] reduces carrier responsiveness to LPS challenge [3]. The polymorphism is associated with unfavourable clinical outcome in several clinically important conditions including septic shock, inflammatory bowel disease (IBD), childhood respiratory syncytial virus infection, and both gastric and colon cancers [5]–[8].

Recognition of LPS occurs via a heterodimeric complex formed between TLR4 and myeloid differentiation factor 2 (MD2). LPS binds to the large hydrophobic pocket in MD2 to induce the formation of an m-shaped receptor multimer comprising at least two copies of the TLR4-MD2-LPS complex [9]. The TLR4 Asp299Gly polymorphism lies within the extracellular domain of the receptor. Crystallography work shows that in the crystal structure of LPS bound TLR4 Asp299Gly/MD2 forms receptor dimers in the same way as wildtype TLR4 [10], but there are local conformational changes. The effects of the Thr399Ile polymorphism on the LPS-TLR4/MD2 structure are minimal. Despite a body of excellent crystallographic and functional studies precisely how these SNPs alter TLR4 reactivity remains unclear.

Upon ligand binding the TLR4/MD2 receptor complex ultimately recruits the adaptor proteins myeloid differentiation primary-response protein 88 (MyD88) and TIR-domain-containing adaptor protein-inducing IFN-B (TRIF). MyD88 dependent signalling activates IKK (IκB kinase) and mitogen-activated protein kinase (MAPK) pathways. The IKK pathway, through regulation of early phase NF-κB, controls expression of proinflammatory cytokines and other immune related genes [11]. MAPK pathway activation induces another transcription factor AP-1 which also plays a role in proinflammatory cytokine expression [12], [13]. NF-κB activation with delayed kinetics also occurs in the absence of MyD88 directed signalling [14] through TRIF and TRIF-related adaptor molecule (TRAM). This pathway also activates transcription factor interferon regulatory factor 3 (IRF3) to induce Type I interferons [15], [16]. Factors which alter the balance between these signalling pathways will impact on the immune response including appropriate adaptive immune cell recruitment [17]. The effect of the TLR4 Asp299Gly polymorphism on adaptor protein recruitment is controversial. Analysis of human genetic data has led to the hypothesis that the TLR4 Asp299Gly polymorphism may primarily signal through TRAM/TRIF rather than MyD88 which would alter the cytokine profile of patients with this SNP [18], [19]. Recent work using TLR4^−/−^ mouse macrophages expressing human TLR4 Asp299Gly polymorphism suggest there is a deficit in both MyD88 and TRIF recruitment by the mutant receptor, although these experiments were performed in the absence of human MD2, meaning that complete receptor function was not permitted [20].

The cosegregating polymorphism, Thr399Ile (rs4986791), in TLR4, affects the functional consequences of the Asp299Gly polymorphism. Early studies by Arbour and Schwartz showed that carriers of the Asp299Gly polymorphism had decreased NF-κB activity compared with wildtype TLR4 [3], [21]. Later work showed that carrying the TLR4 Asp299Gly polymorphism alone was associated with increased disease risk and increased TNF-alpha production in comparison to having wildtype or the Thr399Ile polymorphism [18]. The *TLR4* Asp299Gly polymorphism therefore has important implications for disease outcome, yet the precise mechanisms by which this SNP has its effects remain undefined.

Here we show, for the first time, that TLR4 carrying Asp299Gly and Thr399Ile polymorphisms have blunted responses to LPS compared to wild-type TLR4 as expected but, these constructs also have high levels of constitutive activity in reconstituted signalling assays. Unstimulated monocytes from patients carrying these mutations, compared to patients with wild-type TLR4, have downregulated expression of several genes in the TLR4 TRAM/TRIF signalling pathway. LPS stimulation of monocytes from patients carrying the TLR4 mutations biases TLR4 signalling through the TRAM/TRIF pathway. Taken together these data suggest that the Asp299Gly and Thr399Ile polymorphisms lead to TLR4 having some level of basal activity altering the expression of genes in the TRAM/TRIF signalling pathway partially explaining why upon LPS stimulation there is a dysregulated inflammatory response.

## Results

### Presence of the TLR4 Asp299Gly and Thr399Ile polymorphisms drives altered basal TLR4 signalling

**Figure 1:**
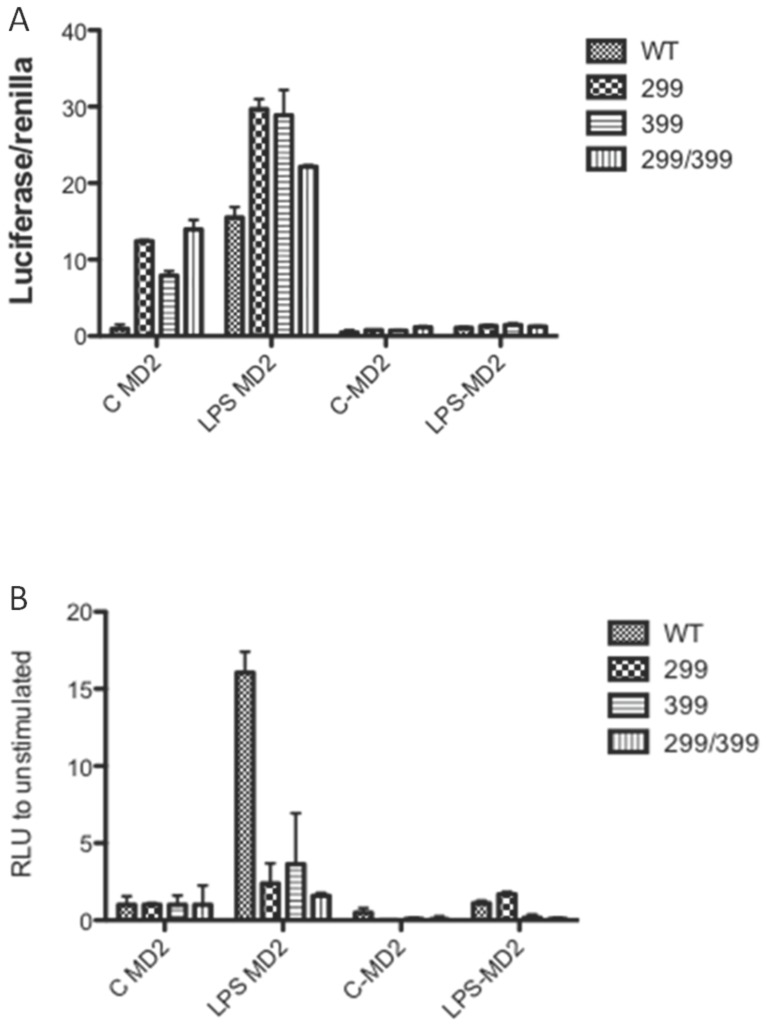
Transient transfection of TLR4 Asp299Gly, TLR4 Thr399Ile or TLR4 Asp299Gly/Thr399Ile with MD2 into HEK cells results in constitutive NF-κB activation but a reduced relative increase compared to basal NF-κB activity. HEK cells were transiently transfected with wild-type or mutant TLR4 +MD-2 +CD14, together with the reporter constructs NF-κB-luc and Renilla luciferase. After 48 h the cells were stimulated for 6 h with medium alone (C) or medium +10 ng/ml LPS (LPS) and the luciferase activity was determined in cell lysates. Data are from a representative experiment (n = 3 experiments) and are shown as mean ±SEM for that experiment. A) Relative luciferase activity of wild type TLR4, Asp299Gly TLR4, Thr399Ile TLR4 or Asp299Gly/Thr399Ile TLR4 with or without stimulation with 10 ng/ml LPS. B) Fold increase in luciferase activity compared to basal activity induced by stimulation of wild type TLR4, Asp299Gly TLR4, Thr399Ile TLR4 or Asp299Gly/Thr399Ile TLR4 with 10 ng/ml LPS. C MD2  =  unstimulated cells in the presence of MD2; LPS MD2  =  LPS stimulated cells in the presence of MD2; C-MD2  =  unstimulated cells with MD2 absent; LPS-MD2  =  LPS stimulated cells with MD2 absent.

To study the impact of the polymorphisms on TLR4 signalling two approaches were used. We first transiently transfected wild-type human TLR4, TLR4 Asp299Gly, TLR4 Thr399Ile or TLR4 Asp299Gly/Thr399Ile along with human MD2 and CD14 into HEK cells and determined the effects of these receptor constructs on basal and LPS-induced NF-κB activation. We were surprised to find that each TLR4 mutant, unlike native TLR4, elicited a level of basal activity in unstimulated cells. This basal activity was detected only in the presence of MD2 indicating that this constitutive receptor activity required the complete TLR4/MD2 complex (Figure 1a). When stimulated with LPS the fold increase in activity over control for each of the mutants was markedly reduced compared to the increase in LPS-induced activation of wild type TLR4 (Figure 1b). These results suggested that unstimulated monocyte/macrophages from patients carrying the TLR4 SNPs may have basally altered levels of pro-inflammatory gene expression in comparison to cells from patients with wild-type TLR4 if the results of our transient transfection analysis were physiologically relevant. This could result in either increased or decreased pro-inflammatory gene expression given that prolonged stimulation of TLR4 can result in an “endotoxin tolerance” state whereby pro-inflammatory gene expression is down-regulated.

**Figure 2:**
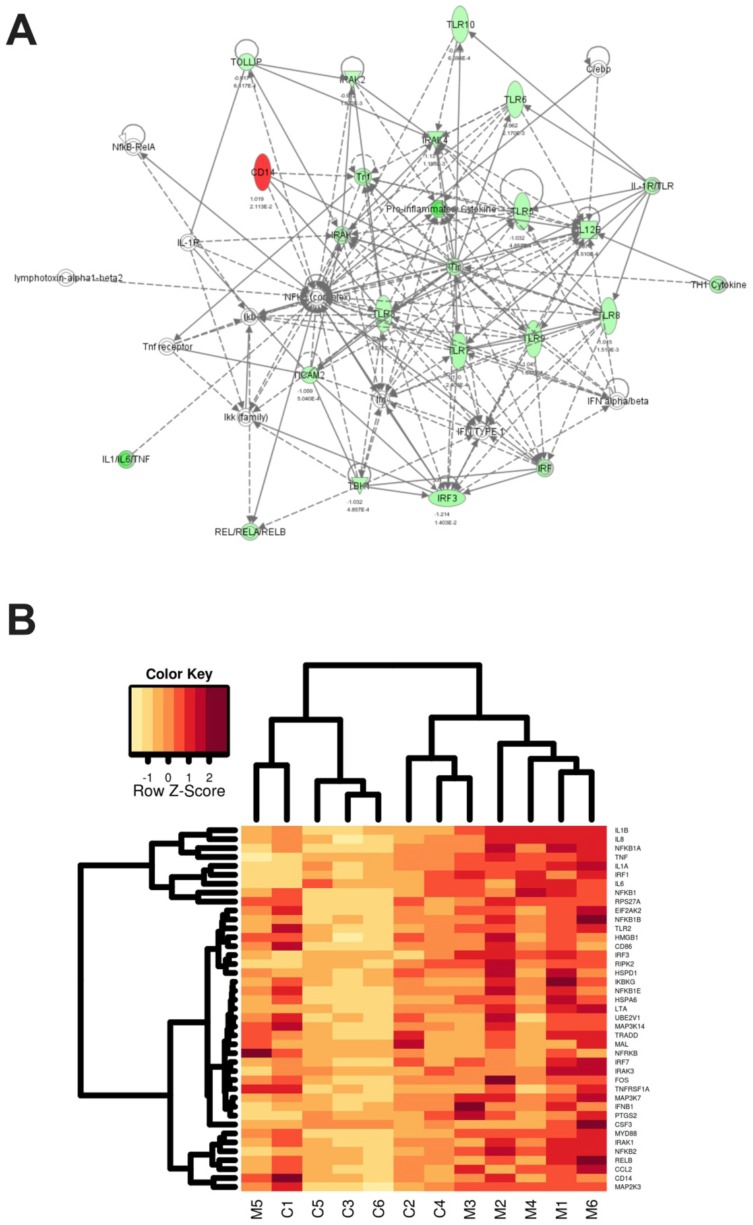
Functional linkage of gene expression patterns. (A) Representation of gene expression patterns affected by the TLR4 Asp299Gly and Thr399Ile polymorphisms in unstimulated PBMCs using Ingenuity pathway analysis. A log_2_ ratio cutoff of 1 was set to focus on genes with greater than 2-fold differential regulation between the two groups. Intensity of colouring indicates the strength of the up-regulation (strong green, highly up-regulated in TLR4 wildtype subjects; strong red, highly upregulated in TLR4 polymorphic subjects; no colour indicates genes included to provide completeness of pathways). (B) Hierarchical clustering analysis of selected gene regulation by the TLR4 Asp299Gly and Thr399Ile polymorphisms in PBMCs following 2 hr LPS stimulation. All genes (n = 41) that following normalisation to housekeeper gene showed down-regulation in polymorphic samples compared to wildtype under LPS stimulation. The columns depict individual subjects (1–6), C =  TLR4 wildtype subjects, M =  TLR4 polymorphic (Asp299Gly and Thr399Ile) subjects. Each row represents a single gene, the key on LHS represents the range of expression level values from the data displayed in the heatmap.

To assess this we conducted a screen of one hundred and fifty healthy volunteers from the North East of Scotland for the presence of the Asp299Gly and Thr399Ile polymorphisms. Fourteen individuals (9.3%) were heterozygous for the TLR4 Asp299Gly polymorphism of which 11 (7.3%) were also heterozygous for the Thr399Ile polymorphism. No homozygous carriage of either polymorphism was observed and only 3 individuals (2%) carried the single Asp299Gly allele. This is in line with previous observations relating to individuals of European descent [6], [22]. A cohort of 10 TLR4 polymorphic carriers (heterozygous carriage of both Asp299Gly and Thr399Ile polymorphisms) was selected for further analysis along with 10 roughly sex and age matched TLR4 wildtype volunteers (who did not carry the TLR4 Asp299Gly and Thr399Ile polymorphisms). We performed gene expression profiling analysis on RNA derived from unstimulated PBMCs to identify target genes specifically affected by the presence of the TLR4 genetic variants. We used the Oligo GEArray Human Toll-Like Receptor Signaling Pathway Macroarrays which comprises 113 genes central to TLR-mediated signal transduction and innate immunity. Using normalisation to housekeeping genes (GAPDH and HSPCB) we performed functional linkage of gene expression patterns using pathway analysis which showed that the majority of genes were downregulated in the TLR4 polymorphic carriers compared to the TLR4 wildtype subjects (92 out of 113; Figure 2a). Although not all of these differences were statistically significant, this is considerably more than the 6 genes that would be expected purely due to chance. To reduce the number of false positives we applied an additional foldchange cutoff of 2 (1 on the log2-scale). Twenty one of the 113 genes investigated were identified as having consistently altered gene expression (greater than 2 fold increase or decrease) between wild type and TLR4 polymorphic carriers with a statistical significance level of P<0.05 including TLR5, 7, 8 and 9 (Table 1). In general higher levels of gene expression were seen in TLR4 wildtype individuals with 20 genes showing increased expression and only CD14 gene expression showing higher expression levels in TLR4 polymorphic carriers (Figure 2a). These findings indicate that healthy individuals carrying the TLR4 Asp299Gly and Thr399Ile genetic variants have altered basal immune gene activity under normal conditions compared to TLR4 wildtype subjects.

**Table 1:** Log ratio values for TLR4 polymorphic/wild type gene expression of components of the TLR signalling pathway under basal conditions.

Gene symbol	Protein name	Log_2_ mean ratio TLR4 polymorphic/Wild type (respective fold change)
**MyD88 dependent genes**		
IRAK4	Interleukin-1 receptor-associated kinase 4 (IRAK4)	−1.13
MAP3K7IP1	TGF-beta-activated kinase 1 and MAP3K7-binding protein 2 (TAB1)	−1.02
NFKBIL2	Tonsoku-like protein (IKBR)	−1.00
PTGS2	Prostaglandin G/H synthase 2 (COX-2)	−2.11
RELA/p65	NF-κB subunit p65	−1.02
RIPK2	Receptor-interacting serine/threonine-protein kinase 2 (RIP2)	−1.35
SITPEC	Evolutionarily conserved signalling intermediate in Toll pathway (ECSIT)	−1.00
**Trif dependent genes**		
IRF1	Interferon regulatory factor 3 (IRF1)	−1.10
IRF3	Interferon regulatory factor 3 (IRF3)	−1.21
TBK1	Serine/threonine-protein kinase TBK1	−1.03
TICAM2	TIR domain-containing adapter molecule 2 (TRAM)	−1.06
**Non pathway specific genes**		
CD14	Monocyte differentiation antigen (CD14)	+1.02
CSF3	Granulocyte colony-stimulating factor (G-CSF)	−2.97
IL-12B	Interleukin 12B	−1.07
IL2	Interleukin 2	−1.2
PGLYRP3	Peptidoglycan recognition protein 3	−1.05
TLR5	Toll like receptor 5	−1.03
TLR7	Toll like receptor 7	−1.11
TLR8	Toll like receptor 8	−1.02
TLR9	Toll like receptor 9	−1.04
UBE2N	Ubiquitin-conjugating enzyme E2	−1.03

A value of −1 is equivalent to a 2 fold increase in gene expression in wildtype subjects compared to TLR4 polymorphic subjects. A value of +1 is equivalent to a 2 fold decrease in gene expression in TLR4 polymorphic subjects compared to wild type. All fold changes presented were statistically significant with p value <0.05.

### Carriers of the TLR4 Asp299Gly and Thr399Ile polymorphisms have dysregulated LPS-induced TLR4 signalling

To determine the effects of the TLR4 Asp299Gly and Thr399Ile genetic variants on induction of the TLR4 downstream signalling response, we stimulated monocytes with LPS and derived RNA from healthy wildtype volunteers and healthy TLR4 polymorphic carriers. We performed further gene expression profiling to look for LPS induced gene expression differences between wildtype and TLR4 genetic variant-carrying individuals. We used hierarchical clustering of the array data and included genes that were downregulated in TLR4 variant carriers. It was evident that following 2 hr LPS stimulation, the presence of the TLR4 Asp299Gly and Thr399Ile variants was associated with decreased gene expression in many genes compared to wildtype individuals (Figure 2b). The hierarchical analysis clustered the 12 subjects into two groups with four of the TLR4 wildtype subjects clustering with one of the TLR4 polymorphic subjects in cluster one. Cluster two contained two sub-clusters: a cluster of 4 TLR4 polymorphic subjects and a second mixed subject group cluster comprising the remaining two TLR4 wildtype subjects and the other TLR4 polymorphic subject. Although the hierarchical analysis was not able to definitively cluster subjects based on TLR4 genotype, there was clear evidence of gene expression differences indicating that carriers of the TLR4 Asp299Gly and Thr399Ile polymorphisms were responding differently to LPS challenge compared to TLR4 wildtype subjects. Forty-five genes were shown to have at least 2 fold difference in gene expression between the wildtype and TLR4 Asp299Gly and Thr399Ile polymorphism carriers with statistical significance level of P<0.05 (Table 2). All genes showed decreased expression in the TLR4 Asp299Gly and Thr399Ile polymorphism carriers.

**Table 2:** Log ratio values TLR4 polymorphic/wild type following stimulation with *E. coli* LPS.

Gene symbol	Protein name	Log_2_ mean ratio TLR4 polymorphic/Wild type (respective fold change)
**MyD88 dependent genes**		
CHUK	Inhibitor of nuclear factor kappa-B kinase subunit alpha (IKK-α)	−1.14
IRAK2	Interleukin-1 receptor-associated kinase 2 (IRAK2)	−1.17
IRAK4	Interleukin-1 receptor-associated kinase 4 (IRAK4)	−1.21
MAP2K4	Mitogen-activated protein kinase kinase 4	−1.22
MAP2K6	Dual specificity mitogen-activated protein kinase kinase 6	−1.20
MAP3K1	Mitogen-activated protein kinase kinase kinase 1	−1.08
MAP3K7IP1	TGF-beta-activated kinase 1 and MAP3K7-binding protein 2 (TAB1)	−1.29
MAP3K7IP2	TGF-beta-activated kinase 1 and MAP3K7-binding protein 2 (TAB2)	−1.10
MAP4K4	Mitogen-activated protein kinase kinase kinase kinase 4	−1.11
MAPK10	Mitogen-activated protein kinase 10	−1.09
MAPK12	Mitogen-activated protein kinase 12	−1.13
MAPK14	Mitogen-activated protein kinase 14	−1.19
NFKBIL2	Tonsoku-like protein (IKBR)	−1.33
PTGS2	Prostaglandin G/H synthase 2 (COX-2)	−1.37
RELA/p65	NF-κB subunit p65	−1.10
SITPEC	Evolutionarily conserved signalling intermediate in Toll pathway (ECSIT)	−1.33
TOLLIP	Toll interacting protein	−1.27
**Trif dependent genes**		
PELI1	E3 ubiquitin-protein ligase pellino homolog 1	−1.08
SARM1	Sterile alpha and TIR motif-containing protein 1	−1.23
TBK1	Serine/threonine-protein kinase TBK1	−1.34
TICAM2	TIR domain-containing adapter molecule 2 (TRAM)	−1.32
TRIF	TIR domain-containing adapter molecule 1	−1.16
**Non pathway specific genes**		
CLECSF9	C-type lectin domain family 4 member E	−1.24
CSF3	Granulocyte colony-stimulating factor (G-CSF)	−2.50
CXCL10	C-X-C motif chemokine 10	−1.03
HRAS	GTPase HRas (p21)	−1.17
IFNG	Interferon-γ	−1.24
IL2	Interleukin 2	−1.19
IL10	Interleukin 10	−1.00
LY86	Lymphocyte antigen 86	−1.13
NR2C2	Nuclear receptor subfamily 2 group C member 2	−1.19
PGLYRP1	Peptidoglycan recognition protein 1	−1.11
PGLYRP2	Peptidoglycan recognition protein 2	−1.17
PGLYRP3	Peptidoglycan recognition protein 3	−1.30
PGLYRP4	Peptidoglycan recognition protein 4	−1.18
PPARA	Peroxisome proliferator-activated receptor alpha	−1.17
PRKRA	Interferon-inducible double stranded RNA-dependent protein kinase activator A	−1.29
TLR1	Toll like receptor 1	−1.15
TLR3	Toll like receptor 3	−1.29
TLR5	Toll like receptor 5	−1.35
TLR6	Toll like receptor 6	−1.23
TLR8	Toll like receptor 8	−1.29
TLR9	Toll like receptor 9	−1.07
TLR10	Toll like receptor 10	−1.09
UBE2N	Ubiquitin-conjugating enzyme E2	−1.30

A value of −1 is equivalent to a 2 fold increase in gene expression in wildtype subjects compared to TLR4 polymorphic subjects. All fold changes presented were statistically significant with p value <0.05.

**Figure 3:**
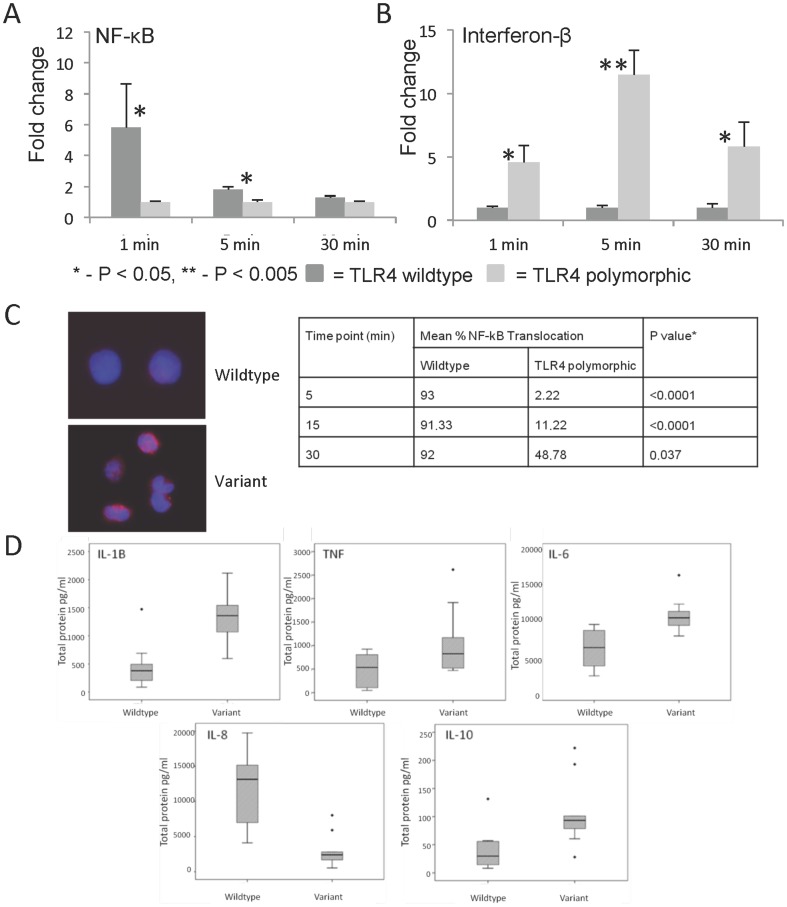
TLR4 Asp299Gly and Thr399Ile polymorphisms have dysregulated LPS-induced TLR4 signalling. (A and B) TLR4 polymorphisms affect gene expression in both the MyD88 dependent and independent signalling pathways. NF-κB and IFN-B gene expression following *E. coli* LPS stimulation (1 µg/ml) depicted as fold change after normalisation to unstimulated values. Graphical representation reflects fold change difference between median values from wildtype and TLR4 polymorphic carriers. Error bars  =  Standard error. (C) TLR4 polymorphisms inhibit p65-phosphorylation Human PBMCs were incubated with or without *E. coli* LPS (1 µg/ml) upto 30 mins (15 min LPS stimulation depicted) and then analysed for the distribution of p65 by immunofluorescence. Red stain indicates the localization of p65, and blue stain indicates the nucleus (magnification, 200x). Mean percentage of NF-κB translocation in peripheral blood derived monocytes from wildtype individuals and carriers of the TLR4 Asp299Gly polymorphism. Data generated from 3 individuals per group tested in 3 separate experiments. *T-test statistic for comparison at each time point between wildtype and TLR4 variant carriers. (D) TLR4 polymorphisms affect downstream cytokine production. Box-and-whisker plots of cytokine production by monocytes from wildtype and TLR4 Asp299Gly and Thr399Ile polymorphic carriers stimulated with *E. coli* LPS (1 µg/ml) for 24 h. The **bold lines** represent medians, and the span of the box represents the interquartile range of the data, * depicts outliers. Data is generated from sample groups of 10 volunteers.

The hierarchical clustering identified a number of cytokine genes as differentially expressed which indicated that more changes in the response to LPS were potentially occurring further upstream and may have been missed at the 2 hr timepoint. To examine this more fully, genes from both the MyD88 dependent and independent signalling pathways were selected for further study. Again using the monocyte/LPS challenge system, gene expression was compared between the two groups, although particularly focussing on the early phase response i.e. <1 hr. This demonstrated that NF-κB gene expression levels were increased in all subjects following LPS stimulation, however consistent with the transfection studies they were significantly higher in wildtype individuals compared to TLR4 polymorphic carriers (Figure 3A). Following *E. coli* LPS stimulation, the TLR4 wildtype group demonstrated >6 fold higher levels of NF-κB gene expression compared to TLR4 polymorphic carriers (P<0.05; MWU test). This significant increase was also seen at 5 minutes although the difference between TLR4 wildtype and polymorphic carriers was reduced with equivalent levels detected by 30 minutes. This altered NF-κB gene expression coincided with increased expression of IFN-β in TLR4 polymorphic carriers (Figure 3B). In contrast to NF-κB gene expression, IFN-β levels were raised following LPS stimulation in TLR4 polymorphic carriers whereas no difference in IFN-β gene expression was detected in TLR4 wildtype carriers when LPS stimulated levels were compared to unstimulated levels (Figure 3B). The TLR4 polymorphic carriers showed significantly increased levels of IFN-β gene expression at all time points studied compared to wildtype subjects, with the maximal difference detected following LPS stimulation for 5 mins where ∼12 fold difference in IFN-β gene expression levels were detected between TLR4 polymorphic and wildtype subjects.

The effect of the TLR4 polymorphisms on the nuclear retention of NF-κB (p65) was also examined by immunocytochemistry. Monocytes were stimulated with LPS for up to 30 minutes upon which time they were fixed and double stained with anti-p65 antibody and DAPI. Fixed cells were observed under fluorescence microscopy and the percentage of cells showing NF-κB translocation was recorded. NF-κB translocation was significantly slower in TLR4 polymorphic subjects (Figure 3C). There was no significant difference in the level of NF-κB translocation across all time points studied in the wildtype subjects (p = 0.693; Welchs ANOVA). However there was a significant difference in NF-κB translocation in TLR4 polymorphic subjects (p = 0.02; Welchs ANOVA). The results suggest that NF-κB was less readily activated with delayed kinetics in the presence of the polymorphisms (Figures 3A and 3C) consistent with additional signalling occurring through the Tram/Trif pathway. This difference in NF-κB translocation time was also accompanied by altered inflammatory cytokine production. Comparison was made between basal/unstimulated cytokine production and following LPS stimulation. The TLR4 polymorphic carriers showed a significant increase in production of IL-1β, TNF-α, IL-6 and IL-10 and significantly lower IL-8 levels after LPS stimulation compared to wildtype subjects. These differences were not detectable at earlier time points (Figure 3D: 24 hr data shown). In general, the response of PBMCs from healthy volunteers that carried the polymorphisms were significantly altered compared to the wildtype subjects which ultimately resulted in significantly increased cytokine levels following LPS stimulation.

## Discussion

Our work shows, for the first time, that the TLR4 Asp299Gly and Thr399Ile polymorphisms confer altered constitutive activity upon the TLR4 receptor and this is associated with basal down-regulation of many pro-inflammatory genes in unstimulated monocytes from patients carrying these SNPs. Our data is consistent with a model whereby monocytes from patients carrying the SNPs have an elevated level of constitutive TLR4 receptor activity resulting in suppression of basal pro-inflammatory gene expression similar to what is seen in endotoxin tolerance models. The basal activity of the TLR4 Asp299Gly and Thr399Ile particularly effects genes in the TRAM/TRIF, rather than the MAL/MYD88, signalling pathway. This suggests that in monocytes from patients carrying the SNPs the constitutive TLR4 activity is either not activating or switching off MYD88 signalling whilst TRIF signalling is conserved. This is consistent with data where TLR4 stimulation with monophosphoryl lipid A results in less PI3K-dependent recruitment of MYD88 to the TIR therefore reducing signalling through this pathway whilst leaving fully functional TRAM/TRIF signalling [23]. Alternatively the polymorphisms may inhibit the formation of higher order complexes such as the Myddosome that are required for MyD88 directed signalling [24]. It is, however, unclear how the SNPs induce constitutive TLR4 activity but presumably there is a conformational change which results in altered TLR4/MD2 dimerisation, TIR dimerisation to recruit adaptor proteins and/or intracellular trafficking to the endosome to facilitate TRIF signalling.

LPS stimulation of the TLR4 SNPs in HEKs resulted in small increases in NF-κB activation over basal TLR4 activity in comparison with wild-type TLR4. Data from transfection studies are usually presented as fold increase in NF-κB activity over unstimulated control thus an elevated baseline NF-κB activity would blunt any fold increase of the LPS-induced response. This is consistent with blunting of the LPS-induced response through activation of TLR4 Asp299Gly and Thr399Ile polymorphisms compared with wild type TLR4 seen previously [3]. In functional studies on monocytes the TLR4 Asp299Gly and Thr399Ile polymorphisms have a significant impact on TLR4 downstream signalling. In these cells LPS induces less NF-κB activation and biases downstream signalling in favour of the TRAM/TRIF pathway in comparison to wild-type TLR4. Several studies have reported the effects of polymorphism carriage, usually by reporting cytokine levels, but our work suggests a molecular explanation for the differences detected. We suggest it is not the magnitude of the immune response that is affected but more that the polymorphisms are changing the pattern of TLR4 adaptor protein recruitment. Recent work where the human TLR4 SNPs were expressed in murine macrophages showed reduced recruitment of both MYD88 and TRIF to the mutant TLR4 signalling complex [20]. The work is partially, but not completely, consistent with our results. The differences between this work and our own may well be because we are studying patient cells heterozygote for mutant and wild-type TLR4 where the receptor is endogenously expressed in the presence of human MD2. Understanding the differences in heterozygote carriers is of most clinical relevance as the polymorphisms are present in around 8–10% of the Caucasian population with the majority of carriers being heterozygote. The other main difference stems from the fact that the Figueroa study did not include human MD2 in their murine macrophage experimental setup. Mouse MD2 does not confer fully signalling capacity to human TLR4 and results in less efficient signalling such that subtle differences in how human TLR4 behaves maybe lost [25].

Previous studies have presented conflicting data when performing functional studies on cells from patients with the TLR4 polymorphisms. Several *in vitro* experiments have failed to show differences in response between TLR4 Asp299Gly and Thr399Ile polymorphism carriers and wild type subjects [26]–[28]. Many of the studies that failed to show differences in cytokine levels, however, only conducted studies over a short timeframe i.e. <6 hours. Our study showed that cytokine production from cells carrying the TLR4 SNPs was not different from wild-type TLR4 cells in the first 8 hours of LPS stimulation, but differences are seen at later time points, presumably because of the impact of these mutations on the TRAM/TRIF signalling pathway which induces delayed NF-κB signalling. An *in vivo* study by Marsik demonstrated significantly reduced IL-1B levels after 24 hours accompanied by a slight increase in IL-6 levels in TLR4 Asp299Gly polymorphism carriers. It is unclear in this study as to what the TLR4 Thr399Ile status of the polymorphic carriers was. A further PBMC study, in contrast, reported significantly decreased cytokine levels in TLR4 polymorphism carriers [29]. In this study a combined polymorphic group containing individuals with either or both of the Asp299Gly or Thr399Ile polymorphisms were used making it difficult to directly compare their data to our own. Our work considered the response of heterozygous carriage of both TLR4 polymorphisms which is the most common haplotype in Caucasian populations and demonstrated that a number of pro-inflammatory cytokines, including IL-1B, TNF-α and IL-6 were expressed at higher levels in TLR4 polymorphism carriers, when assessed after 24 hrs. In our study population, almost all polymorphic individuals (11 out of 14) had both the Asp299Gly and Thr399Ile polymorphisms. Three individuals carried only the Asp299Gly polymorphism and no-one was polymorphic only for Thr399Ile. Homozygous carriage of either polymorphic variant was not detected in our cohort and only individuals that had heterozygous carriage of both functional variants were included in the study.

A study by Ferwerda demonstrated that individuals with the Asp299Gly but not the Thr399Ile polymorphism had a stronger proinflammatory cytokine response reporting increased TNF-α levels following LPS stimulation of blood compared to wild type TLR4 individuals [18]. The study also assessed carriers of both the Asp299Gly and Thr399Ile polymorphisms but did not detect differences compared to the wild-type TLR4 subjects however, the numbers in the polymorphic group were small (N = 4). The conflicting results between the various studies raises the question as to whether the genetic background of a study population can influence the response.

In conclusion TLR4 Asp299Gly and Thr399Ile polymorphisms induce a decreased level of constitutive immune activation which appears to impact on subsequent responses to LPS challenge. Initial blunted responses appear to be followed by exaggerated immune responses suggesting that these variants act in combination with other immune processes to influence the outcome in infectious diseases.

## Materials and Methods

### Research Ethics Statement

The study was approved by North of Scotland Research Ethics Service (05/S0801/128), and written informed consent was obtained from all subjects.

### Plasmid constructs and construction

Wild-type pCDNA3-TLR4 expression vector was provided by Prof E. Latz (University of Bonn, Germany). Point mutations encoding an aspartic acid (Asp) to glycine (Gly) substitution at the amino acid position 299 (Asp299Gly) and a threonine (Thr) to isoleucine (Ile) substitution at amino acid position 399 (Thr399Ile) in human TLR4 protein were created by site-directed mutagenesis using a QuikChange mutagenesis kit (Stratagene) according to the manufacturer's instructions using the following primer pairs: Asp^299^Gly mutation (Asp299Gly) (nucleotide change, A896→G): forward primer: 5′-cttagactactacctcgatgGtattattgacttatttaattg-3′; reverse primer: 5′-caattaaataagtcaataata**C**catcgaggtagtagtctaag-3′; Thr^399^Ile mutation (Thr399Ile) (C→T, nucleotide change, C1196→T): forward primer: 5′- caaagtgattttgggacaa**T**cagcctaaagtatttagatc-3′; reverse primer: 5′-gatctaaatactttaggctg**A**ttgtcccaaaatcactttgagaacag-3′ (nucleotide changes are shown in upper case bold). Complete sequence confirmation of each construct was performed to ascertain effective point mutagenesis had occurred and that no random mutations had been introduced.

### Cells and transfection analysis

HEK293 cells were maintained in Dulbecco's modified Eagle's medium (DMEM) supplemented with 10% fetal calf serum, 2 mM L-glutamine, 100 U.ml^−1^ penicillin and 100 µg.ml^−1^ streptomycin. HEK293 cells were transfected as previously described [30]. Briefly cells were seeded at 3×10^4^/well of a 96 well plate and transiently transfected 2 days later. Expression vectors containing cDNA encoding TLR4 (wildtype and variant forms), MD-2 and CD14 (1 ng/well of each), a NF-κB transcription reporter vector encoding firefly luciferase (5 ng/well pNF-κB-luc, Clontech), and a constitutively active reporter vector encoding Renilla luciferase (5 ng/well phRG-TK, Promega), together with empty vector to ensure an optimal amount of DNA were mixed with JetPEI (Polyplus transfection) according to the manufacturer's instructions. After 48 hours cells were stimulated with KDO2-lipidA diluted in DMEM supplemented with 0.1% fetal calf serum. The cells were washed with PBS and then lysed, and luciferase activity was quantified using the Dual Luciferase kit (Promega) according to the manufacturer's instructions. Luciferase data was analysed using One-Way Analysis of Variance (ANOVA) within SPSS.

### Volunteer recruitment and genotyping of TLR4 Asp299Gly and Thr399Ile polymorphisms

One hundred and fifty healthy male and female volunteers aged between 20 and 55 years of age were recruited from the North East of Scotland to participate in the study. The study was approved by North of Scotland Research Ethics Service (05/S0801/128), and written informed consent was obtained from all subjects. Genotyping of the TLR4 Asp299Gly and Thr399Ile polymorphisms was performed on genomic DNA extracted from leucocytes using pre-designed Applied Biosystems 5′ nuclease SNP genotyping assays, using minor groove binding (MGB) probes 5′-labelled with VIC or FAM (6-carboxyfluoresceine) fluorochromes to detect the wildtype and variant alleles respectively. Allelic discrimination analyses were prepared using standard reactions conditions as described previously [6]. Representatives of the required genotypes were sequenced for definitive confirmation and then taken forward and used for functional studies.

### PBMC studies

PBMCs were isolated by density gradient centrifugation as described previously [31]. PBMCs (>95% viability) were prepared from 10 TLR4 Asp299Gly/Thr399Ile variant carriers and 10 TLR4 wildtype individuals. PBMCs were seeded at 1×10^6^ cells in tissue culture plates. Cells were allowed to adhere for 1.5 h at 37°C in 5% CO_2_. Non-adherent cells were removed by twice washing with PBS. Adherent cells (predominantly monocytes) were stimulated for up to 48 h with medium alone or *E. coli* LPS (1 µg/ml). Culture supernatants were harvested and stored at −80°C until analyzed for cytokine protein levels (TNF-α, IL-1β, IL-6, IL-8 and IL-10) by cytometric bead array (CBA: BD Biosciences). Cells were either fixed for NF-κB translocation as described previously [32] or harvested for total RNA. Immunofluorescence microscopy images for NF-κB translocation studies were taken using a 100×1.4 NA lens on a microscope (IM200; Carl Zeiss MicroImaging, Inc.). Contrast and brightness of individual channels were adjusted linearly in Photoshop (Adobe). Data analysis was undertaken using Welch version of ANOVA.

### Total RNA extraction and gene expression studies

Total RNA was extracted from monocytes using the QIAGEN RNeasy mini RNA extraction kit (QIAGEN, Sussex, UK) according to manufacturer's instructions. First-strand cDNA synthesis was performed in one of 2 ways: for assessment of Toll-like receptor downstream signaling, Oligo GEArray Human Toll-Like Receptor Signaling Pathway Macroarrays (SABiosciences, France) were utilised. cDNA synthesis used 500 ng of total RNA, with 5 µg of the resulting cRNA used for chip hybridisation. Image analysis was performed using a Fuji LAS1000 chemiluminescent detection system and AIDA Image Analyser software (Raytest Isotopenmessgeräte GmBH, Germany). Raw intensities were log-transformed with base 2 and normalised with respect to HSPCB as a housekeeping gene. TLR4 wildtype and TLR4 polymorphic groups were compared with or without *E. coli* LPS stimulation. The comparisons were made using a so-called moderated F-test which was available in the Bioconductor library limma for testing for differentially expressed genes in macroarray experiments. TLR4 group (wildtype or polymorphic) and subject were used as factors in the linear model, so that the test was a moderated version of a paired t-test, and provided us with a p-value and average log2-ratio to assess the difference between wildtype and polymorphic groups. Applied Biosystem gene expression assays were used to validate the macroarray findings. cDNA synthesis was performed using AB High capacity RNA-to-cDNA synthesis kit with between 50 and 100 ng of total RNA used for cDNA synthesis (volunteer dependent). Custom plates were designed which contained the following genes: *AP-1, BTK, CD14, IFNB1, IRAK1, IRAK4, IRF3, LBP, MAP3K7, MAP3K7IP1, MAPK14, MAPK8, MD-2, MYD88, NFKB1, NFKB1A, NFKB1B, REL, RELA, RIPK2, TBK1, TICAM1, TICAM2, TIRAP, TLR2, TLR4, TOLLIP, TRAF3* and *TRAF6*. Gene expression was performed using an Applied Biosystems 7900HT real-time PCR system (Applied Biosystems, Warrington, UK). Relative gene expression of *E. coli* LPS stimulated samples was compared to unstimulated control samples. The comparative cycling threshold method (ΔΔCT) was used for relative quantification after normalisation with GAPDH and HSPCB expression. Statistical analysis was performed using the Mann Whitney U test (MWU test), P values of <0.05 were considered significant.
